# International Symposium on *Angiostrongylus* and Angiostrongyliasis, 2010

**DOI:** 10.3201/eid1707.102038

**Published:** 2011-07

**Authors:** Zongli Diao, Chonghong Yin, Haiyu Qi, Jing Wang

**Affiliations:** Author affiliation: Beijing Friendship Hospital Capital Medical University, Beijing, People’s Republic of China

**Keywords:** Angiostrongylus, angiostrongyliasis, nematodes, foodborne infections, parasites, international symposium, online conference summary

The third International Symposium on *Angiostrongylus* and Angiostrongyliasis was held April 8–9, 2010, at the Faculty of Medicine Siriraj Hospital, Mahidol University, Bangkok, Thailand. Twenty-nine researchers from 7 countries attended the symposium. The symposium’s theme was “Advances in the disease, control, diagnosis and molecular genetics of the genus *Angiostrongylus* and angiostrongyliasis.”

In discussing the epidemiology of the disease, it was noted the first reported case of human angiostrongyliasis came from Taiwan in 1945. Since then, several outbreaks and >2,800 cases of this disease have been reported worldwide. However, the number of cases is probably underestimated given that many physicians might be unaware of angiostrongyliasis because of its rarity; thus, numerous cases might have gone unreported or unrecognized.

Although angiostrongyliasis was mainly prevalent in the Pacific islands and Southeast Asia, increasing numbers of natural foci of the disease have been reported because of increased global trade and travel. Pilar Foronda Rodriguez, University of La Laguna, Spain, reported *Angiostrongylus cantonensis* in *Rattus rattus* in Tenerife, Canary Islands, and confirmed the island as a natural focus of this disease (*1*). Lv Shan, Chinese Center for Disease Control and Prevention, reported natural infection by *A. cantonensis* in 7 provinces in China, including Fujian, Zhejiang, Hunan, Guangdong, Guangxi, and Hainan, and confirmed these provinces as natural foci.

Diagnosis of the infection can be difficult. Recovery of the nematode from an infected patient confirms human angiostrongyliasis. However, the frequency of detecting these nematodes is low. Presumptive diagnosis can be based on clinical symptoms, medical history, and laboratory findings in blood and cerebrospinal fluid. A history of eating intermediate hosts is also crucial for diagnosis of angiostrongyliasis.

In 2006, a total of 160 persons, 100 of whom were hospitalized, were associated with an angiostrongyliasis outbreak in Beijing, China. This finding is comparable with the total number of infections recorded in China over the past decade. Chenghong Yin of Beijing Friendship Hospital, Capital Medical University, China, reported the diagnosis criteria of angiostrongyliasis used during the outbreak. The criteria included the following indices: 1) epidemiology, 2) clinical symptoms, 3) complete blood count, 4) cerebrospinal fluid investigation, 5) immunologic examination, 6) imaging examination, and 7) etiologic examination. Etiologically and clinically positive patients were further defined. Any patient showing larvae was definitively etiologically positive, and displaying indices 1–4 were considered clinically positive, and those with indices 5 and/or 6 were considered to be displaying auxiliary signs consistent with angiostrongyliasis.

In recent years, immunologic detection of this infection has been rapidly developed. Xiaoguang Chen, Southern Medical University, China, purified the major antigenic protein AC32 from adult *A. cantonensis*, and constructed an AC32-ELISA kit to detect specific immunoglobulin (Ig) G in serum samples of patients. AC32 of *A. cantonensis* was subsequently found to be a valuable candidate antigen with high sensitivity and specificity for immunodiagnosis of angiostrongyliasis. Xiaoxian Gan, Zhejiang Academy of Medical Sciences, China, and Praphathip Eamsobhan, Mahidol University, Thailand reported a simple and rapid dot-immunogold filtration assay to detect a specific IgG against *A. cantonensis* that uses crude extracts from adult *A. cantonensis* as the antigen and protein A conjugated with colloid gold as the detection marker. This immunoassay was highly sensitive (90.5%, 19/21) and specific (98.0%, 98/100). However, some cross-reactivity against serum samples from patients with *Trichinella spiralis* infection and schistosomiasis was observed. No cross-reactivity against serum samples from patients with other infections of helminthiasis (e.g., cysticercosis, clonorchiasis, and fasciolopsiasis) or tuberculosis was reported. Therefore, this assay is not only rapid and simple without requiring special instrumentation, but also rather sensitive and specific for the detection of IgG against *A. cantonensis* infection.

There is no specific treatment for angiostrongyliasis. Treatment predominantly relies on symptomatic relief, such as antiinflammatory corticosteroid therapy. Kittisak Sawanyawisuth, Khon Kaen University, Thailand reported a series of studies on the treatment of this disease. In 2000, Sawanyawisuth et al. conducted a prospective, placebo-controlled and double-blind study to assess prednisolone treatment of angiostrongyliasis. Patients in the treatment group were given a 2-week course of prednisolone (60 mg/day), and patients in the control group were given placebo. Results suggested that a 2-week course of prednisolone was beneficial in relieving headache in patients with eosinophilic meningitis. In 2007, a prospective, randomized, double-blind and placebo-controlled study indicated that a 2-week course of albendazole appeared to reduce the duration of headache in angiostrongyliasis. In 2009, they conducted a prospective, randomized and controlled study to compare the efficacy of combined prednisolone/albendazole and prednisolone alone therapies for the treatment of angiostrongyliasis. One hundred four patients were divided into combined treatment and prednisolone alone groups, 53 and 51 patients, respectively. The dosages of prednisolone and albendazole were 60 mg/day taken orally in 3 divided doses and 15 mg/kg/day taken as 2 divided doses after meals for 2 weeks, respectively. However, the results indicated that combined prednisolone/albendazole treatment of patients with angiostrongyliasis was no better than prednisolone alone (*2*). In conclusion, the corticosteroid treatment was beneficial for the patients, although the role of anthelmintic agents remains inconclusive. Therefore, it was not recommended that albendazole alone should be used for the treatment of angiostrongyliasis.

In addition to discussing these recent findings, a cooperative network among researchers and clinicians was established to share data from current and future research projects for the prevention, control and treatment of human angiostrongyliasis. We look forward to the 2012 International Symposium on *Angiostrongylus* and Angiostrongyliasis, which will be held in Guangzhou, China.
